# A fractionated analysis of hot and cool self-regulation in cigarette smokers from different socioeconomic backgrounds

**DOI:** 10.1371/journal.pone.0220222

**Published:** 2019-08-20

**Authors:** Raquel de Luna Antonio, Sabine Pompeia

**Affiliations:** 1 Departamento de Psicobiologia, Universidade Federal de São Paulo, São Paulo, São Paulo, Brazil; 2 Curso de Naturologia, Universidade Anhembi Morumbi, São Paulo, São Paulo, Brazil; University of California San Diego School of Medicine, UNITED STATES

## Abstract

Smoking cigarettes and low socioeconomic status (SES) are both related to impaired cognition. However, it is unknown whether people of lower SES, who comprise most tobacco smokers worldwide, are more susceptible to cognitive impairment associated with smoking. In this non-randomized, cross-sectional study we investigated the effects of cigarette smoking, SES and their interaction on dissociable executive or “cool” and “hot” measures of behavioural self-regulation. Participants (n = 80) were selected among young physically and mentally healthy smokers and non-smokers who had graduated high school and were from different SES backgrounds. Cool self-regulation was measured by executive function tasks that tap inhibition, updating, shifting, dual tasking, planning, access to long-term memory (semantic fluency), and working memory capacity. Hot measures assessed self-reported impulsivity, delay discounting and risk taking. Exposure to tobacco (cotinine, exhaled carbon monoxide, tobacco dependence, cigarette consumption) was assessed to determine to what extent it mediated the cognitive effects of smoking. Nicotine abstinence and its acute effects were controlled, as were sex, age, schooling, and psychiatric symptoms despite the fact that smokers and non-smokers were selected as being as similar as possible in these demographic characteristics. Lower SES (less years of parental schooling) was associated with worse performance on tasks that measured all cool domains except dual tasking and fluency, while smoking status was related to impaired delayed discounting and impulsivity (hot domains), effects that were not mediated by tobacco exposure. Smoking and SES, however, did not interact. In short, impaired performance in measures of most cool skills was associated with SES irrespective of smoking status; in contrast, regardless of SES, smokers showed specific impairment in hot self-regulation domains (more difficulty resisting immediate temptations and weighing future consequences of actions). Possible explanations for the lack of mediation of tobacco exposure on hot skills of smokers are discussed.

## Introduction

A fifth of the world’s population smoke tobacco according to WHO estimates [1]. This habit is more prevalent among socioeconomically disadvantaged individuals, both when comparing smoking rates in developing *versus* developed countries, and when contrasting people of different socioeconomic status (SES) from the same nation [2–4]. Both smoking [5–9] and low SES [10–13] are related to the ability to control or regulate behaviour. It is therefore surprising that the effects of SES on cigarette smokers’ self-regulation have not been explored in the literature, especially as low self-regulation is associated with addictive behaviour and difficulty in quitting smoking (e.g., [14–16]). Our aim was to determine whether dissociable self-regulation cognitive domains are impaired in smokers and whether this pattern of effects is altered by SES.

In order to explain the rationale of the present study we will first address the broad concept of behavioural self-regulation and the fact that it may be fractionated into distinct domains, which could be differently affected by smoking and SES. Next, we will describe the effects of smoking and SES on these separable cognitive abilities, as well as the possible effects of tobacco exposure on behaviour.

### Self-regulation

Behavioural *self-regulation* (henceforth, self-regulation) can be regarded as a broad conceptual construct that embeds a set of limited-resource abilities that enable control/regulation of behaviour (see [17–20]) within a short temporal perspective (see [19]). These abilities enable the adaptation and maintenance of certain behaviours in order to achieve goals set by the individuals themselves and/or actions determined as socially acceptable (see [21]). Self-regulation skills thus encompass executive functions, controlled attention, working memory and skills that regulate thought, emotions, impulses and risk taking [17–19]. These behaviours may be categorized into hot and cool skills. Hot self-regulation involves emotions, beliefs, desires, decision-making, and activities that are rewarded or punished, ultimately emotional regulation and social behaviour [22–24] that are often collectively named impulsivity (e.g. [14,18,25]). In turn, cool self-regulation is not directly related to affect or social experiences and generally includes tasks that tap executive functioning [19,22].

Because the effects of smoking and SES have been associated with impairment in these domains in separate studies, as will be detailed below, it is still unclear whether they affect self-regulation in general, hot or cool skills, or if specific hot and cool domains are susceptible to impairment by these factors. Here we sought to determine which of these abilities are susceptible to these effects and if they interact. Therefore, we examined the literature that speaks to fractionation of these cognitive abilities.

There is still no consensus as to how hot self-regulation abilities should be categorized. The hot domains usually mentioned in the literature [25] involve: 1) selection of immediate smaller reinforcements in detriment of larger ones after a period of time (usually evaluated by Delay Discounting tasks); 2) inhibition of automatic responses that are at odds with task demand, sometimes regarded as cool domain, as will be discussed below; 3) differences in intensity of motivation in relation to internal or external cues with different affective values (which may be measured by tasks that assess risk taking); and 4) individual differences in the general ability to modify or inhibit behaviours in the face of negative consequences, often measured by questionnaires such as the Barratt Impulsiveness Scale (BIS-11 [26]).

Cool self-regulation, in turn, is usually investigated under the term *executive functions*, which also encompass various correlated but dissociable cognitive abilities. At least 6 separable executive domains have been identified (see [27]): 1) *updating–*the efficiency of adding or deleting contents in working memory [28,29]; 2) *shifting* or *switching*, sometimes called *cognitive flexibility–*the ability to alternate between different activities [28,29]; 3) the efficiency of *access to long-term memory* (see [30]), usually assessed using verbal fluency tests; 4) *dual tasking–*the ability to perform simultaneously two tasks that load on different short-term storage domains (see [28,31,32]); 5) *planning*, or the ability to organize behaviour toward a specific goal that must be achieved through intermediate stages [33,34], which is also often included among hot self-regulation skills (see [14,25,35]); 6) *inhibition–*the ability to override automatic, prepotent or dominant responses (see [28,29,36]). Inhibition overlaps with abilities that are common to other cool functions such as maintenance and management of goals during ongoing processing (see [37]). Thus, impairment in inhibition alone could explain worse performance in many other self-regulation skills. Working Memory Capacity, or the ability to keep information in mind in the face of interference (see [38]), is also associated with self-regulation and has been claimed to account for a considerable portion of variance in hot and cool skills (see [19]).

### The effects of smoking tobacco on self-regulation

Shortly after acute administration of nicotine, one of the chemicals in tobacco, both smokers and non-smokers perform better on tasks that assess cool self-regulation in the domains of shifting, attention, and working memory [39–42]. Psychomotor speed is also enhanced [43–45]. Despite the low effect sizes of these effects (see meta-analysis by [43]) they may partly explain the motivation to initiate and maintain smoking habits [43], together with the acute reinforcing effects of nicotine for emotional intensity and mood [46].

Acute effects of nicotine in smokers and non-smokers, however, differ because the fluctuating nicotine concentration in smokers may lead to different cognitive effects depending on when testing takes place. For instance, after smoking a cigarette, the rapid decline in nicotine concentrations in the brain due to its short elimination half-life and distribution to other tissues (see [46]) cause withdrawal symptoms that may impair cognition (e.g. [42]). These effects are evident from around two hours after smoking the last cigarette (see [43]), so it has been claimed that the acute effects of nicotine in abstinent smokers may simply consist of a reversal of abstinence, rather than a beneficial cognitive effect (see [43,47,48]). Furthermore, chronic exposure to nicotine results in tolerance (see [49,50]), which may alter performance and mood and exacerbate psychiatric symptoms [46]. Cigarette smoking has also been found to lead to structural and functional alterations in brain areas related to cognitive functioning [51–53].

Most studies that investigated self-regulation in smokers, however, disregard the differential effects of acute nicotine exposure, nicotine abstinence and the chronic effects of smoking [43]. In these publications, smokers display difficulty in inhibiting behaviour [54,55], are more impulsive [6,9] and more averse to waiting for monetary rewards [56–61]. Higher risk taking in smokers is also found in some studies [7,62], but not most [8,63–66]. All of these measures may be regarded as tapping hot self-regulation. Nevertheless, apart from the absence of control for acute and withdrawal effects of nicotine, the abovementioned effects were found in studies that varied widely in terms of sampling, testing and experimental design, so it is difficult to determine whether smoking affects only hot functions, self-regulation in general or specifically distinct self-regulation domains.

In the few studies that controlled for acute and/or withdrawal effects of nicotine on measures of self-regulation, smokers did not show deficits in Inhibition or Verbal Fluency, but they were more impulsive [9] and showed Planning difficulties [67], suggesting that performance on some, but not all hot abilities are more impaired in this population. Indeed, Fox et al. [68] did not observe associations between smoking and cool executive functions, including Inhibition. Assuming that chronic smoking impairs hot more than cool cognitive abilities makes sense because the former are related to emotional/motivational responses that lead people to smoke and have difficulty quitting (e.g., [14–16]). However, this intuitive association may have biased researchers towards prioritizing the study of hot self-regulation in this population.

Mitchell [14] suggested three possible non-exclusive explanations for smokers’ hot self-regulation problems. The first is that people who become smokers are those with the worst self-regulation and therefore more vulnerable to smoking (see [9]). Another possibility is that people who find it difficult to self-regulate cannot stop smoking once they have started. These predictions have been confirmed (e.g. [9,67,69]). Another explanation is that continuous tobacco exposure causes biological changes [49,50,70] that alter behaviour. In this case, measures of tobacco exposure should mediate the effects of smoking on self-regulation.

The reasons given by Mitchell [14] for the relationship between hot self-regulatory impairment and smoking may, of course, also apply to cool domains (see [67,71]). Any attention/executive difficulty could increase people’s propensity of become smokers, given that acute nicotine doses improve performance in these domains (see [67]), or result from exposure to tobacco, despite conflicting evidence [9,67] that may, however, reflect the fact that only a small set of parameters related to this exposure was investigated.

In order to further the understanding of what tobacco smoking does to cognition and what can be done to help smokers regulate or cease this habit, studies should identify which aspects of self-regulation are affected by chronic smoking in the same experimental setting, controlling for acute and withdrawal effects of nicotine. To this end, we investigated the effects of chronic cigarette smoking in all the above mentioned self-regulatory domains, avoiding most acute effects of nicotine and nicotine withdrawal (see the Methods section for details).

### The effects of socioeconomic status on self-regulation

SES is a complex multidimensional construct that encompasses economic measures, power and prestige, and which is modified by family and social experiences [72,73]. Lower SES during childhood negatively impacts cerebral development [74] and self-regulation abilities later on in life. These effects seem to be related to low cognitive stimulation (e.g. reading) [75]. Studies in this field, however, have mostly focused on cool domains—Inhibition, Updating, Planning and Working Memory Capacity (see [71,76,77]) -, in contrast to the higher prevalence of studies that assess hot cognition in smokers. Bickel et al. [71] suggest that individuals with low SES display impaired cool executive functions that negatively influence their ability to control emotions and impulses. Inzlicht et al. [78], on the other hand, suggest that all measures that tap self-regulation involve resolution of conflict, and are therefore similar regardless of the hot or cool nature of the tasks. Nevertheless, reports of a relation between low SES and poor hot self-regulation are rare and inconsistent [79–81].

### The present study

Because of the high association of smoking and low SES, our aims were to better characterize the self-regulatory profile of smokers by assessing many hot and cool self-regulation skills and to determine whether performance on these measures was influenced by SES. The sample consisted of young healthy adults from diverse socioeconomic backgrounds, in whom smoking-induced self-regulation effects can be isolated from those of aging, health problems and use of psychotropic medication, which are more prevalent as people age. Studying this population is essential if we are to formulate strategies to increase adherence to treatment because early cessation of this habit and/or reduced smoking may decrease cognitive impairment caused by smoking [5], morbidities and mortality [82]. Various mediators of tobacco exposure on self-regulation were also considered: nicotine metabolite cotinine [83], self-reported dependence to nicotine, daily and lifetime cigarettes smoked (see [9]), as well as exhaled carbon monoxide. We hypothesized that smokers’ self-regulation would be generally impaired, and that lower SES smokers would be more susceptible to these negative effects of smoking because of the deleterious effects of lower SES on self-regulation.

## Materials and methods

### Participants

Eighty healthy participants aged 18 to 35 years with at least 11 years of schooling took part in this study. Smokers were defined as people who has smoked at least 10 cigarettes per day in the previous year and who had smoked daily for at least two years (half the sample). The remainder were non-smokers (control group) who were also not passive smokers [i.e., had not lived with smokers or shared a working environment in which people could smoke indoors] because environmental exposure to cigarette smoke may impact cognition [82]. Non-smokers, however, could have smoked up to a maximum of 100 cigarettes in their lifetime, because there is evidence that consumption of more cigarettes can be related with neuronal reward pathway functioning (see [84]). All participants had body mass index (BMI kg/cm^2^) between 19 and 29 (obese participants were excluded because this condition may interact with SES and affect cool self-regulation [85]) and had normal or corrected to normal vision. They reported no history of hearing problems, learning disabilities, or diagnosis of neuropsychiatric or clinical illnesses. The participants were not on medication at the time of the study and did not consume alcohol or illicit drugs (cannabis) more than twice a week.

### Procedure

All experimental procedures were approved by the Ethics Committee of Universidade Federal de São Paulo (#131.810). Written informed consent was obtained from all participants, who were recruited through e-mails, posters or direct personal contact at the University where the study took place and in surrounding neighbourhoods.

Four hundred and twenty-five people responded to the screening questionnaire, which contained health and demographic questions including various measures of SES (see below), as well as the Self-Reporting Questionnaire, which assesses psychiatric symptoms (SRQ-20; [86]) and the Fagerström Test for Nicotine Dependence (FTND; [87]). After many exclusions, only 80 potential participants (40 smokers) met the eligibility criteria, a little short of our predicted sample size (see details in the section of Statistical Analyses below). The other individuals were not included mainly because they smoked fewer than 10 cigarettes a day (n = 79), had BMI over 30 (n = 50), were taking psychoactive medication (n = 22), or were passive (n = 9) or were on-smokers who had been former smokers (n = 8). Almost a quarter of potential volunteers (n = 97), the great majority of whom were smokers, were excluded due to SRQ score indicative of psychiatric disorders (see Discussion section for possible reasons for this). Because somatic symptoms are more prevalent in smokers [88], we decided to include participants with SRQ scores up to 11 [86,89].

This was a non-randomized, cross-sectional study. Only participants who reported having kept to their pre-test routine in terms of adequate sleep were tested, always individually during the morning. Females were tested during the first four days of their menstrual cycle in order to homogenize their hormonal status, which may influence mood, cognition [90–92], smoking behaviours [93] and central effects of psychoactive drugs [94].

As soon as participants arrived, smokers were asked to smoke one of their own cigarettes in the presence of the experimenter. They were only allowed to smoke again after the end of the experiment. Because nicotine concentrations decline rapidly 20 min after finishing a cigarette [95], testing began 30 minutes after this cigarette in order to exclude most acute effects of nicotine. Non-smokers also waited 30 min after arrival before being testing. The test battery lasted less than 90 min so that abstinence to nicotine would not interfere with results [43,96]. Participants had their transportation expenses paid.

The test battery commenced with mood assessment using visual-analogue scales. The hot and cool self-regulation measures were then applied in four different pseudo-random orders, balanced between participants and groups (smokers and non-smokers) so that fatigue could not account for our findings. To prevent participants developing strategies to carry out the tasks, tests were performed only once (see [97]), in all cases preceded by practice trials to endure that subjects had understood the instructions. All stimuli were visually presented (see [5]) because of possible smoking-induced impairment in auditory processing, comprehension, and receptive vocabulary. Importantly, we adapted the cool executive tasks to ensure they would not contain stimuli that could be better processed by people with better verbal/auditory competence due to better schooling [98]. Therefore, we only included stimuli (words or pictures) that are recognized by young children [99], and altered stimuli such as letters to numbers because better schooled/non-smoking participants could have more knowledge of acronyms, which could aid performance. At the end of the test session the participants were tested on the variables associated with tobacco exposure (urine samples for the analysis of cotinine and exhaled carbon monoxide). Other non-self-regulation measures were also obtained and are reported elsewhere (e.g., buccal swab to evaluate cytotoxicity: [100]).

### Measures

#### Socioeconomic status

Socioeconomic background: because there is no consensus on the best measure of SES we collected data on mothers’ and father’ years of schooling separately [76,101], *per capita* family earnings [102], purchasing power (family assets [103]), and work prestige of the main breadwinner [104]. Using a composite score of all these SES measures would complicate interpretation of our findings and increase the difficulty in replicating our results. Hence, we chose to select only one variable as a proxy of SES in our analyses to investigate the possible interaction with smoking status. This SES variable was selected based on its association with general knowledge, know to reflect prior cognitive stimulation throughout people’s lives (Information test, see below).

Information task (from the Wechsler Adult Intelligence Scale, WAIS-III, [105] adapted for local use [106]): consists of 28 general knowledge questions and ends after six consecutive mistakes. The dependent measure is the proportion of correct answers. Scores in this task are regarded as measures of general knowledge, which correlates with schooling and SES (see [21,107,108]).

Performance in this task best related to parent’s average number of years schooling among all SES measures parental schooling was used to index SES in all our analyses (see the Results section).

#### Exposure to tobacco (mediators of effects of smoking on self-regulation)

Cigarette consumption: the number of cigarettes smoked per day at the time of the experiment was computed, as well as the number of cartons of cigarettes smoked in a lifetime (one carton = 10 packs of 20 cigarettes each, or 200 cigarettes), estimated by multiplying the number of cigarettes smoked per day by the number of years of cigarette smoking.

Fagerström Test for Nicotine Dependence (FTND; [87] adapted for local use [109]): a self-reported measure consisting of 6 questions that evaluate dependence on nicotine, with scores ranging from 0 (no dependence) to 10 (highest dependence).

Exhaled carbon monoxide: measured using the monoximetre Smokerlyzer (Bedfont) device. Participant are asked to inhale and retain air for 10 s, after which they exhale into a disposable mouthpiece for as long as possible. The carbon monoxide values are determined in parts per million (ppm).

Cotinine excretion: midstream urine was used to analyse cotinine concentrations (ng/mL). The collector pot was kept on ice until samples were processed (within 30 min). Five mL of urine were centrifuged at 3000 rotations per minute for 15 minutes and 1.5 mL were stored in an Eppendorf tube in a freezer at -80°C. Samples were analysed as per the immunoassay kit manual (ABNOVA—KA0930).

#### Mood measure

Self-Reporting Questionnaire **(**SRQ-20; [86], adapted for local use [110]): this self-report scale includes 20 questions that assess non-psychotic mental disorders symptoms, rated on “yes” or “no” answers. The score is obtained by adding a point for each question answered positively. Larger scores indicate a higher prevalence of symptoms of mental disorders. The cut-off point considered internationally and in Brazil is 8 [110], but can be extended to 11 [89].

Visual-Analogue Mood Scale (VAMS; [111] adapted for local use [112]): this questionnaire includes 16 x 100 mm-long visual-analogue scales, each of which represents the full range of a type of subjective dimension (e.g., "sleepy___alert"). Participants are instructed to make a vertical line at the point along each scale that best corresponds to how they feel at that moment, considering ends of scales as the minimum and maximum on each dimension. Scores are the number of mm from the left end of each scale. Four mood domains [112] were considered: anxiety, mental and physical sedation and other symptoms.

#### Behavioural measures

**"Hot" self-regulation measures** Risk taking, assessed by the Balloon Analogue Risk Task (BART; [113]): on a computer screen, a series of 30 balloons were sequentially shown. Each balloon could be inflated by keyboard presses, each of which provided participants with hypothetical 5 cents added to their “bank statement”. Participants were told that they could stop inflating a balloon at any time and cash the total value acquired for that balloon, but if the balloon exploded they would lose all the money acquired for that particular balloon. Participants were also told that they would never know when the balloons might explode. The dependent measures were: a) 'BART score', defined as the average number of inflated balloons that did not explode [113]; b) the Total Number of Explosions; c) Total Collected Cash Amount; and d) 'Cost-Benefit Ratio' [114], or the number of balloons that exploded divided by the total number of balloons presented (i.e. 30), divided by the relative proportion of 'benefit' obtained, that is, discounted total monetary value divided by the maximum possible monetary value that could be earned (i.e. "96 dollars"). In other words, this ratio is equal to the percentage of risk divided by the benefit percentage. A ratio greater than 1 indicates relatively higher risk incurred for less financial benefit, while a ratio of less than 1 indicates relatively greater overall benefit for the level of risk incurred [114].

Delayed Discounting, assessed by inter-temporal monetary decision-making task [115]: this task consists of 21 questions in which the participant is asked if he prefers to earn a hypothetical monetary amount today (x) or a larger amount (x + y) in the future. Monetary rewards and the delays are functions in the scoring calculation: K = ($ future—$ today) / (wait in days * $ today)—$ future. The monetary amounts presented in Brazilian currency resembled those of the original study in US dollars and were divided into low, medium or high reward categories.

Impulsivity, assessed by the Barratt Impulsiveness Scale (BIS-11 [26], adapted for local use [116]): this scale includes 30 questions, such as "I say things without thinking" and "I get bored easily when I am solving problems mentally", answered on 4-point Likert scales. The total score ranges from 30 (lowest impulsivity) to 120 (highest impulsivity). Three dimensions of trait impulsivity were also evaluated [26]: a) motor, related to the non-inhibition of incoherent responses to the context; b) attentional, related to fast decision making; and c) planning: encompasses behaviour oriented to the present.

**“Cool” self-regulation measures** Updating [117], assessed by the Number Memory task (adapted from the Letter Memory task [117,118]): the original task of remembering sequences of letters was adapted to recalling sequences of single digits, presented serially for 2000 ms each. The task consists of recalling the last three numbers presented in the list. To ensure that the task involved continuous updating, the instructions require participants to rehearse the last 3 digits out loud by mentally adding the most recent number and dropping the 4th number back. For example, if the numbers presented are ‘‘7, 3, 5, 9, 2” the participants should say, ‘‘7 … 7 3 … 7 3 5 … 359 … 592 and answer “592” at the end of the trial. After practicing for 3 trials with 5 and 7 digits each, participants performed 12 trials (four sequences each of 5, 7 and 9 digits). List length varied randomly across trials to ensure that participants continuously updated their memory representations until the end of each trial. The dependent measures were the number of trials in which the last three digits were recalled in the same serial order as presented, and errors in oral updating.

Shifting [28], assessed by the Category Switch task (adapted from [36,119]): in each trial of this task participants are presented with an image on the screen which can be classified in terms of two independent semantic contents: size (bigger or smaller than a soccer ball) and as living or non-living objects. The 16 images selected from Cycowicz et al. [120] were adapted from the words used by Mayr and Kliegl [119] and had high naming consistency in children and adults in Brazil (see [121]). Four images were big inanimate objects (mountain, house, ferris wheel, circus) or small ones (clothes pegs, button, key, whistle), and four big living beings (elephant, bear, giraffe, gorilla) or small ones (butterfly, ladybug, ant, worm). The first trial involved judging whether images represented “non-living” or “living” objects. In the second trial participants decide whether the images represented big or small objects. The participants are asked to give their answers aloud and press a key to pass on to the next image. Both these trials included 20 images each, which were randomly mixed regarding semantic contents. The third trial included 40 images and involved alternating semantic characterization while keeping order in mind, with no external cues. Time and errors for the trial completion were measured. Shifting cost was calculated by subtracting the sum of the time to complete the first and second trials from that for the alternating trial. The same was calculated for number of categorization errors.

Dual tasking, assessed by the Dual Task Paradigm [97]: this paper and pencil task includes a visuospatial tracking task (circle crossing) and a phonological task (digit span). First, participants’ digit spans are determined. To do so lists of an increasing number of digits are read aloud at the rate of one digit per s. The participants are asked to repeat these numbers in their order of presentation. The participants’ digit spans are the maximum length at which they correctly repeated 5 of 6 sequences of digits. Once spans are determined, participants are required to repeat sequences that correspond to their spans for 90 s for the phonological task. Scores are the number of sequences repeated correctly divided by the number of sequences presented. In the circle crossing task, the subjects use a pencil to follow a path (319 circles linked with lines that formed a chain) laid out on an A3-sized sheet of paper as quickly and as accurately as possible for 90 s. Scores are the number of circles crossed. There was a training path that served as practice for this task. The dual task condition consisted of the simultaneous execution of both tasks within 90 s. Scores are measured with the Mu index [122].

Access to Long-Term Memory, assessed by the Semantic Verbal Fluency task [30,123]: in this task the participants are given 60 s to orally generate as many words as possible that belonged to two categories (four-footed animals and fruit). The participants were instructed not to use morphological variations of words and to avoid repetitions. The mean number of correct responses and errors for both categories were determined. To control for differences in verbal speed (see [124]**)** we measured how long it took for participants to count from 1 to 50 as quickly as possible.

Working Memory Capacity [38], assessed by the Counting Span task [38]: this complex span task measures the ability to store information in mind while performing a processing task. Arrays consisting of three to nine dark blue circles, one to nine dark blue squares, and one to five light blue circles are randomly displayed onscreen. Participants count out aloud the number of dark blue circles on each array and proceed to the next screen by pressing the space bar immediately after counting. The arrays are presented in blocks which vary randomly in number from 2 to 6. At the end of each block, participants must recall the number of dark blue circles counted in each of the presented arrays in the same order in which they appeared. Scores (all or nothing load scores; ANL) were the sum of the correctly recalled sequential stimuli when all the counts were recalled in correct serial order divided by the total number of items; see [38].

#### Measures of domains considered as both “cool” and “hot” self-regulation

Planning [34], assessed by the Zoo Map Task [125]: this is a paper and pencil task in which participants are given a map of a zoo (with images and symbols instead of names of places as in the original test) and a set of instructions relating to places they have to visit and rules they must follow (instructions and rules were transformed into drawings and diagrams to facilitate the task for low-SES participants). There are two trials with identical aims that involve drawing the path on the map that includes a visit to 6 out of the 12 possible locations. The first trial consists of a “high demand” version in which participants must plan and draw the path of their visit on the map following the established rules. The second trial is a “low demand” version in which the participants are simply required to draw their path on the map following instructions to reach specific locations. In both trials participants can freely consult the instructions and rules. Scores were planning time plus drawing time on the high demand task minus drawing time on the low demand task [126]. Another outcome was the total raw score [34], or number of locations visited in the correct order with points deducted if an error, such as inappropriate places visited, is made (maximum score 16).

Inhibition of prepotent responses [28], assessed by the Stroop test [127] (adapted for local use [128]): this task consists of a list of 24 common words printed in different colours (green, pink, blue, brown) on a single sheet of paper and another similar list with 24 colour names written in incongruent colours (e.g., the word “blue" written in pink ink). The participant must name the ink colours as quickly as possible in both sheets. The time taken and errors are recorded. The score is the reading time of the first list (naming speed) subtracted from the second list (inhibition), giving a measure of the extra time needed to avoid the incongruity of word and ink colours in the second list (inhibition cost). The same is calculated for errors.

### Statistical analysis

Determining the sample size of the present study was not straightforward. We could not predict means and variability in performance from the literature as no study has previously determined the effects of chronic smoking across variable SES on the cognitive variables of interest in the age range of our healthy sample, let alone in a developing country and controlling for acute nicotine effects. To estimate the number of participants we would need to detect cognitive changes with at least large effect sizes we used GPower [129] considering *a priori* analysis with covariates (ANCOVAs as it does not compute information for GLM), two groups (smokers and non-smokers) and two continuous predictors (SES and one other, on average, which we believed might be significant even though we selected smokers and non-smokers to be as alike as possible). This resulted in a total sample size of 84, with critical F = 3.96 and actual power of 0.95.

The descriptive analysis included means and standard deviations of all variables. Inferential analyses mostly involved univariate General Linear Models (GLMs). For all analyses, the level of significance was 5%. Still, in the results section significant and near significant effects (p values <0.07) are reported.

Firstly, to determine the similarity between the groups of smokers and non-smokers, we used Chi squares to compare the proportion of men and women smokers and non-smokers, and Univariate GLMs for each of the other demographic characteristics.

To determine the SES measure that was the best indicator of prior cognitive stimulation that could affect self-regulation, we ran univariate GLMs using the measure of general knowledge from the Information task (WAIS-III, [105]) as the dependent variable and, in separate univariate models, included each of the SES measures as an independent variables. To facilitate interpretation of these exploratory data, the results are shown with the explanatory value (R^2^) of each model, indicating the extent to which each SES measure explained the variance in performance on the Information test. The selected SES measure was averaged parental years of schooling (see below).

To assess changes in self-regulation, univariate GLMs were run separately for each domain and included the factors smoking status (smokers, non-smokers), the selected SES measure (mean parental schooling; continuous predictor) and the interaction of these factors, along with other control (covariate) variables known to influence cognition: age, sex, BMI and number of psychiatric symptoms from the SRQ. The GLM of the Semantic Verbal Fluency task also included verbal articulation speed as a covariate. When no interaction of smoking and mean parental schooling (SES) was found, the interaction was removed from the analysis and other similar GLMs were run to determine the effects of smoking and SES with the other abovementioned control variables. When there were statistically significant effects of the covariates concomitantly with the effect of smoking and/or mean parental schooling (SES), these will be described. When not, the covariates were removed from the models to reduce possible type I errors (see [130]). Therefore, in the cases in which there was no interaction and none of the covariates were statistically significant, we reported the results of the models with smoking and mean parental schooling (SES) as independent factors, since these were the measures of interest. The normal distribution of standardized residues was verified [131] for each statistical model in its final version. Deviant values (lower or higher than three SD) were withdrawn from the analyses. *Post hoc* analyses, when applicable, were carried out using Tukey's Honest Significant Difference tests, which corrects for multiple comparisons.

In the cases in which the dependent measures included several levels, GLMs were also used but with another factor added to the models. This happened when analysing results from the Delayed Discounting test (the dependent factor included three levels: monetary values high, medium and low), the Balloon Analogue Risk Task (three levels: blocks with 10 balloons each), the Barratt Impulsiveness Scale (three levels: motor impulsiveness, attentional and non-planning), and the VAMS (four levels: mood factors).

Effect sizes will be shown in the form of multiple determination coefficients (R^2^), which indicate the proportion of variance of the dependent measure explained by the models. We directed more attention to findings with R^2^ values of 0.13 to 0.25, considered medium effect sizes, and those above 0.26, regarded as large effect sizes [132] to avoid focussing on statitical artifacts. Beta coefficients (B) of each significant variable was also described to facilitate interpretation of findings: there is a one-unit increase in the dependent variable for every increase (positive betas) or decrease (negative betas) in the beta coefficient values.

Mediation analyses were carried out using the SPSS additional package Process [133] with bootstrapping based on 10.000 resamples [134]. For cognitive measures that were affected by SES or smoking, with at least medium effect sizes, we explored whether: 1) the SES effects on each cognitive measures was mediated by other cognitive abilities that are associated with the affected dependent variable (e.g. working memory capacity as a mediator of the effect of SES on planning abilities; more details on the selected mediators are provided in the Results section); and 2)the smoking effect on each cognitive measure was mediated by variables related to tobacco exposure. For each smoking-associated cognitive effect we tested each of the following in separate mediation analyses: self-reported dependence to nicotine, daily and lifetime cigarettes smoked, cotinine and exhaled carbon monoxide. Controls for covariates found to influence the association of SES or smoking with cognition were also included in the mediation analyses. Significant mediation was found when bootstrap confidence intervals did not include zero.

## Results

**Fig 1:**
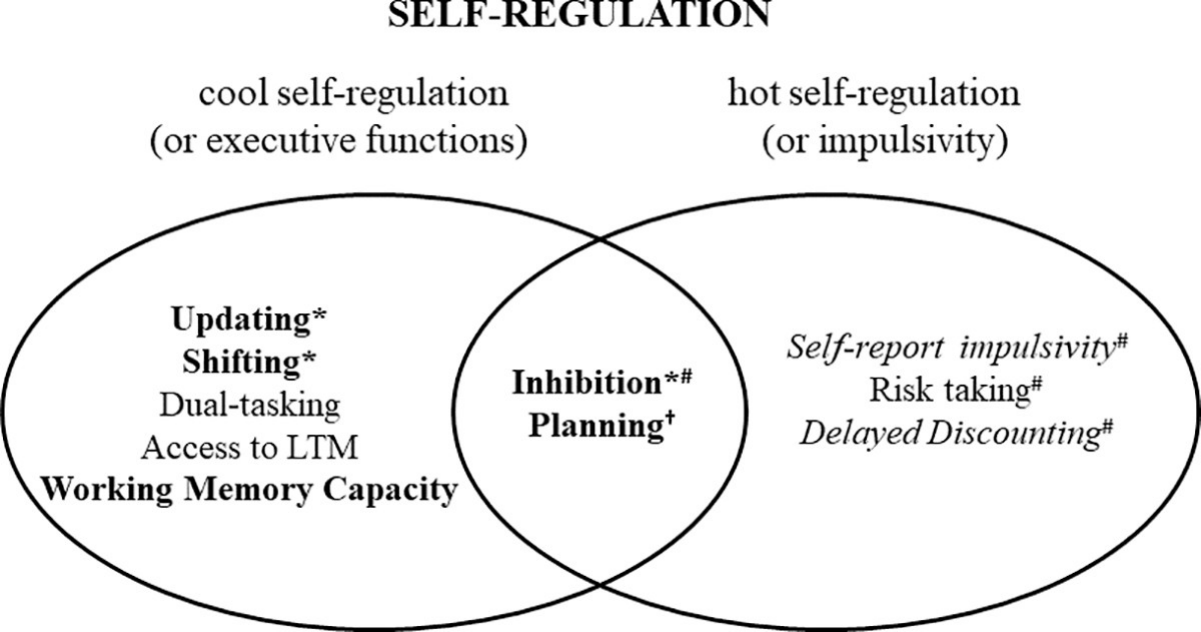
Fractionation of self-regulation of behaviour including hot (impulsivity), cool (executive functions) and intersecting domains which were affected by smoking (in italic) and socioeconomic status (SES; in bold). There was no interaction of tobacco smoking and SES. The rationale for classifying tasks into domains was obtained from [27]; [28]*; [25]^#^; [14]^†^; [19]; [18]^†^.

Data were missing for one volunteer in the BART and two participants in the VAMS scale. In some tests, deviant effects were observed in the GLM normal residues, generally of different participants: one participant each in Category Switch, Stroop, Counting Span, and Dual Task, two participants in the BART, and three in the Zoo Map test. In all these cases the removal of these participants did not alter the pattern of effects, so results were reported including them. Results shown below refer separately to mean parental schooling (SES) and smoking effects, as no interactions between these factors were found for any of the variables (p values> 0.07). Fig 1 illustrates the conception employed here of hot and cool fractionated functions as being part of the broader concept of self-regulation of behaviour, including Inhibition and Planning as intersecting abilities, and the effects of smoking and SES on these abilities, which did not interact.

### Socioeconomic status measures as a proxy for prior cognitive stimulation

Table 1 shows the explanatory values of the various measures of SES when predicting prior cognitive stimulation as measured by the General Knowledge task. Average parental schooling (which ranged from 0 to 20.5 years) had the best explanatory value (21%) so this was the SES measure used for the following analyses.

**Table 1:** Explanatory values [R^2^, and significance values (p)] of univariate general linear models (GLM) in which general knowledge (performance in the Information test) was the depend variable and socioeconomic status measures (SES) were the independent variables, each of which included in a separate model.

Socioeconomic status variable	Statistical results
*Per capita* family earnings (Brazilian Reais)	R^2^ = 0.03; p>0.10
Mother’s schooling (years)	R^2^ = 0.12; p<0.001
Work prestige of the main breadwinner [104] (score)	R^2^ = 0.15; p<0.001
Purchasing power [135] (score)	R^2^ = 0.17; p<0.001
Father’s schooling (years)	R^2^ = 0.20; p<0.001
**Parents' average years of schooling (years)**	**R**^**2**^ **= 0.21; p<0.001**

Note: R^2^ indicates the extent to which the SES measures were associated with general knowledge or, more specifically, which percentage of variance of performance in this task is explained by each SES measure. The **variable in bold**, parents' average years of schooling, was used as a measure of SES indicative of prior cognitive stimulation because of its higher R^2^.

### Effects of socioeconomic status

GLMs with significant effects were univariate and included age of participants, sex, BMI and SRQ scores as control variables, but these covariates were dropped when they did not reach statistical significance. Lower mean parental schooling (SES) was associated with more Updating errors (R^2^ = 0.11, B = -0.21, F_1,77_ = 8.62, p<0.01), higher Shifting cost in errors (R^2^ = 0.13, B = -0.26, F_1,76_ = 11.14, p<0.01) and Inhibition cost in errors (R^2^ = 0.11, B = -0.21, F_1,75_ = 6.42, p<0.02), all with small effect sizes. Planning cost (time) measured with the Zoo Map test was impaired in lower-SES participants (medium effect size R^2^ = 0.18, B = -4.12, F_1,73_ = 5.64, p<0.03). This was the only SES effect that was also affected by a covariate, in this case, age (older participants had higher planning costs: R^2^ = 0.18, B = 4.03, F_1,73_ = 3.90, p<0.01). Worse Working Memory Capacity (R^2^ = 0.22, B = 0.02, F_1,76_ = 14.38, p<0.01) was also found in lower-SES participants (medium effect size).

**Fig 2:**
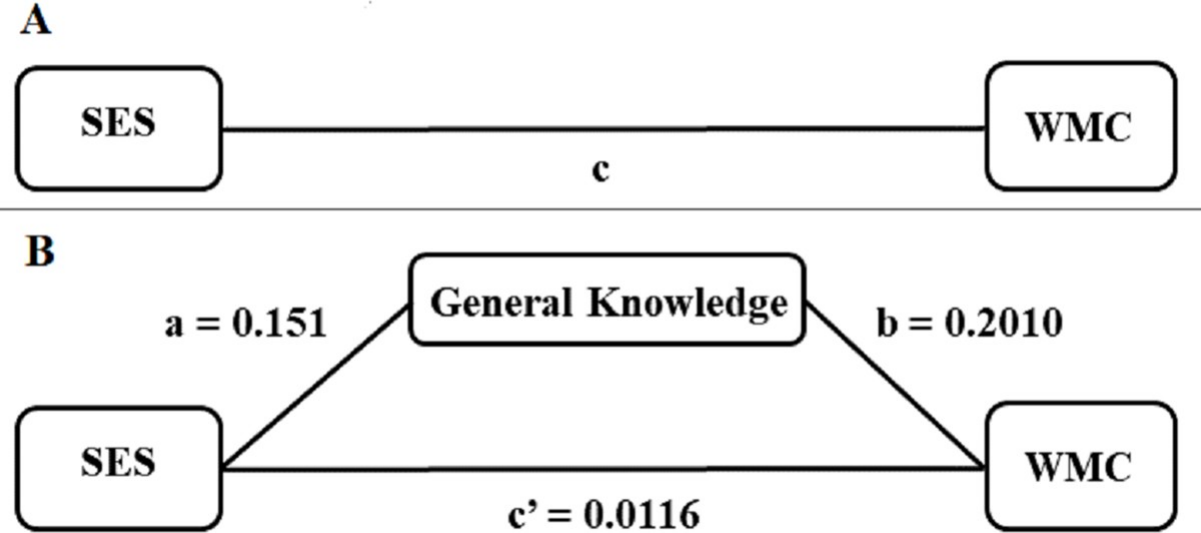
Example of a Mediation model used: In this case, the significant mediation of the effects of parental schooling, our measure of socioeconomic status (SES) on Working Memory Capacity (WMC) by General Knowledge (Information test). Note: A) c is the total effect of the relation between parental schooling and Working Memory Capacity; B) Mediation model; a (path from parental schooling to General Knowledge) and b (path from General Knowledge to Working memory Capacity) constitute the indirect path from parental schooling to Working Memory Capacity; c’ = is the direct path relating parental schooling and Working Memory Capacity.

Mediators of mean parental schooling (SES) effects on Planning were explored, corrected for age: 1) General Knowledge, because it reflects cognitive stimulation during the lifetime (see [21,107,108]); 2) Inhibition (cost of errors in the Stroop test) because it overlaps with a common factor underlying various executive domains [37]; 3) Working Memory Capacity (Counting Span task ANL score), as it is claimed to account for a considerable portion of variance in hot and cool self-regulation skills (see [19,38]). None of these variables significantly mediated Planning. We also explored whether the effect of mean parental schooling (SES) on Working Memory Capacity was mediated by General Knowledge and Inhibition, using the rationale described above. Only General Knowledge partially mediated this effect (Fig 2; route coefficients: a = 0.0151; b = 0.2010; c = 0.0146; c’ = 0.0116; Indirect effect = 0.0030; Standard Error = 0.0018; Confidence Interval Bootstrapping = 0.0003–0.0076) (Fig 2), indicating that General Knowledge was related to both the dependent and independent variables and explained around 20% of the total association between parental schooling and Working Memory Capacity (difference between c and c’).

### Effects of smoking

Regarding smoking status (Table 2), smokers and non-smokers did not differ in terms of sex (chi^2^_(1) =_ 3.27, p = 0.07), years of schooling, mean parental schooling (SES), BMI or mood at the beginning of the test session. Smokers, however, were slightly older than non-smokers (univariate GLM: R^2^ = 0.09, B = -1.56, F_1,78_ = 7.50, p<0.01), and had higher scores on the Self-Reporting Questionnaire (SRQ) (univariate GLM: R^2^ = 0.08, B = -0.89, F_1,78_ = 7.06 p<0.01) (small effect sizes). As expected, smokers had higher concentration of urine cotinine [univariate GLM: R^2^ = 0.92, B(non-smokers) = -51.42, F_1,77_ = 890.38, p<0.01] and exhaled carbon monoxide [univariate GLM: R^2^ = 0.70, B(non-smokers) = -5.82, F_1,77_ = 180.75, p<0.01] than non-smokers (Table 2).

**Table 2:** Characterization of smokers and non-smokers according to demographic variables, mood at the beginning of the cognitive test battery and measures of tobacco exposure post-test (at the end of the test battery).

	Non-smokers (n = 40; 21 ♀)		Smokers (n = 40; 13 ♀)	
	Mean	SD	Mean	SD
Age (years)*	24.43	5.42	27.55	4.77
**Schooling (years)**	15.50	3.25	15.03	2.81
**Body mass index (kg/cm**^**2**^**)**	22.59	2.61	23.70	3.04
**SES (parent’s average schooling; years)**	12.60	4.28	11.64	4.52
**General knowledge (proportion correct)**	0.60	0.20	0.63	0.19
Self-Reporting Questionnaire (score)*,†	2.85	2.77	4.63	3.20
**Visual Analogue Mood Scale (VAMS) levels**				
**- Physical Sedation (score)**	31.25	13.09	24.84	12.80
**- Mental Sedation (score)**	36.14	19.22	35.56	19.88
**- Anxiety (score)**	25.65	17.02	26.20	19.11
**- Other symptoms (score)**	16.60	13.19	14.85	12.20
**Length of smoking behaviour (years)**	0.00	0.00	10.71	5.52
**Current quantity of cigarettes/day (n°)**	0.00	0.00	15.18	4.21
**Time smoking this quantity (years)**	0.00	0.00	6.23	4.86
**Cartons (200 cigarettes) smoked in life (n°)**	<0.5^**#**^	-	2922.3	1744.6
**Fagerström Test for Nicotine Dependence (score)**	0.00	0.00	4.78	1.64
Exhaled carbon monoxide (ppm)*	3.28	1.62	14.80	5.20
Cotinine (ng/mL)*	3.37	9.40	106.20	19.37

Note

*effects of smoking (ps<001)

**†**7 of the 80 participants had scores between 8–11, 5 of whom were smokers

^**#**^23 individuals had never smoked a single cigarette, 8 had smoked up to 5 in their lifetime, 7 had smoked between 6 and 20 cigarettes and 2 had smoked from 21 to 100 cigarettes.

In contrast to the effects of average parental schooling (SES) on most cold self-regulation measures, smoking effects were only found in two hot measures: BIS-11 (large effect size) and Delay Discounting (medium effect size) (Table 3). Importantly, there were no significant interactions between smoking and this SES measure on any variable (p values >0.11).

**Table 3:** Mean (±SD) scores on hot and cool self-regulation measures according to smoking status.

	Non-smokers (n = 40)		Smokers (n = 40)	
	Mean	SD	Mean	SD
**Hot Self-regulation Measures**				
Barratt Impulsiveness Scale				
- Total (score)*	59.05	6.90	66.58	9.53
- Motor (score)*	19.48	2.73	22.70	3.60
- Attentional (score)	17.98	3.20	19.23	3.70
- Non-planning (score)*	22.75	3.66	26.03	4.36
Delay Discounting				
- total (score *k*)	0.04	0.04	0.07	0.03
- low monetary values (*k* score)	0.04	0.04	0.04	0.02
- medium monetary values (*k* score)	0.04	0.05	0.06	0.04
- high monetary values (*k* score)*	0.07	0.08	0.15	0.09
Balloon Analogue Risk Task				
Average number of inflated unexploded balloons	35.98	11.02	32.52	12.02
- balloons 1 to 10	30.33	13.30	28.15	13.19
- balloons 11 to 20	38.69	12.16	33.33	12.40
- balloons 21 to 30	41.01	13.30	37.32	15.77
Total Number of Explosions (n°)	8.62	3.62	7.90	3.80
- balloons 1 to 10	2.33	1.44	2.53	1.43
- balloons 11 to 20	3.15	1.31	2.58	1.47
- balloons 21 to 30	3.13	1.66	32.80	1.56
Total Collected Cash Amount ($)	3764.74	936.44	3382.00	839.23
- balloons 1 to 10	1120.26	423.30	978.88	317.18
- balloons 11 to 20	1295.00	329.22	1165.50	323.27
- balloons 21 to 30	1355.38	348.28	1242.00	373.82
Cost-benefit ratio [114]	0.007	0.003	0.007	0.003
**Cool Self-regulation Measures**				
Category Switch task: Shifting cost (time; s)	33.65	16.62	36.05	15.66
Category Switch task: Shifting cost (errors; no.)	3.10	3.05	3.80	3.98
Number Memory task: Updating (recalled no.)	29.58	5.73	29.03	5.68
Number Memory task: Updating (errors; no.)	2.75	2.94	3.15	2.78
Dual Task: Dual tasking (mu index)	93.10	13.35	90.94	10.03
Fluency: Access to long-term memory (mean no. words/category)	19.70	4.65	20.70	3.94
Counting Span task: Working Memory Capacity (ANL score) [38]	0.394	0.163	0.420	0.190
**Hot and Cool Self-regulation Measures**				
Stroop task: Inhibition cost (time; s)	4.25	3.95	5.00	3.19
Stroop task: Inhibition cost (errors; no.)	0.40	1.20	0.20	0.85
Zoo Map task: Planning cost (time; s)	109.15	55.87	155.70	125.43
Zoo Map task: Planning cost (total raw score)	13.73	3.10	14.23	2.73

Note

* effects of smoking (p<0.05).

**Fig 3:**
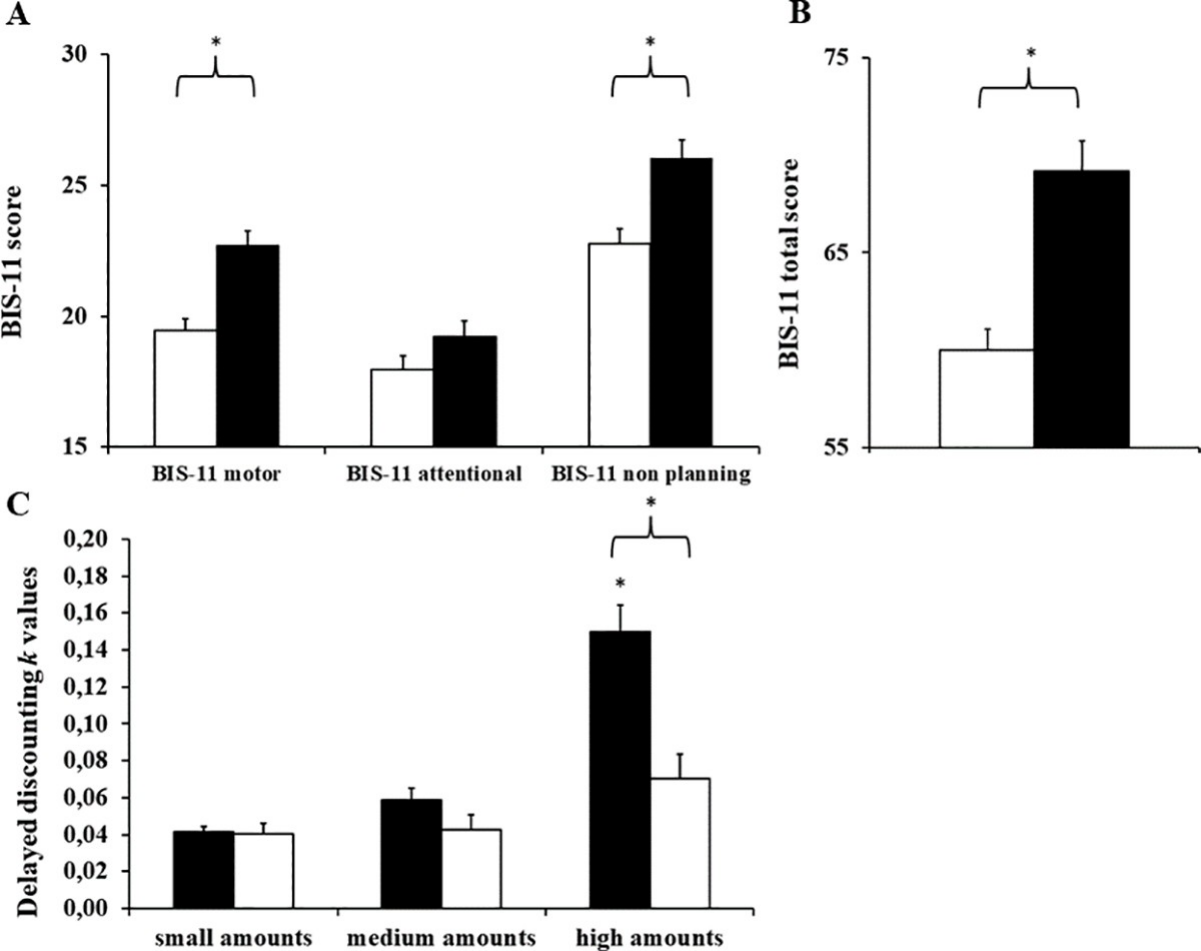
Self-regulation effects of smoking. BIS-11 = Barratt Impulsiveness Scale; BIS-11 motor, attentional and non-planning scores separately (A); BIS-11 total score (B); Delayed discounting *k* values for small, medium and large amount (C). * smokers had worse scores than non-smokers, and, for large monetary rewards, higher than all comparisons (p≤0.05, with medium to large effect sizes).

For the BIS-11, the dependent variables were entered as a factor with three levels (Motor, Attentional and Non-Planning impulsivity). There was an interaction of the impulsive domain with smoking (F_2,150_ = 3.08, p<0.05) in the statistical model that was corrected for factors that influenced BIS-11 scores (higher scores in the Self-Reporting Questionnaire and larger BMI were associated with more impulsiveness): non-smokers reported lower Motor Impulsiveness (R^2^ = 0.26, B = -1.26) and Non-planning Impulsivity (R^2^ = 0.27, B = -1.01) compared to smokers (Tukey *post hoc* ps <0.01) (Fig 3), but were equivalent regarding Attentional impulsiveness.

There was also an interaction between the Delay Discounting dependent factor (three levels: low, medium and high K or financial values) and smoking (F_2,154_ = 16.12, p<0.01). Smokers were more averse to waiting for monetary values when values were large (R^2^ = 0.17, B = 0.0001) than all other conditions among smokers and non-smokers, for whom different monetary values did not affect delay discounting scores (Tukey *post hoc* p values <0.01) (Fig 3).

Cotinine and exhaled carbon monoxide correlated with estimated amount of cigarettes consumed daily (cotinine r = 0.34; p = 0.03; CO r = 0.57, p<0.01). Estimated cigarettes smoked in a lifetime also correlated with CO (r = 0.39, p = 0.01). However, none of these biological measures, nor dependence to nicotine and cigarettes smoked, significantly mediate the effects of smoking on self-regulation variables that were sensitive to smoking (total BIS-11 and Delay Discounting).

## Discussion

Contrary to our hypothesis, we did not find an interaction between the negative effects of smoking and low SES on self-regulation, effects that have been reported separately in the literature [17,78,136,137]. SES, as measured by average parental years of schooling, was associated with changes in most of the assessed cool functions (except dual-tasking and fluency), while smoking was related with performance on most hot functions (impulsiveness and delay discounting, but not risk taking). This double dissociation shows that both the hot and cool measures of self-regulation used here were sensitive to impairment, although distinct factors (smoking and SES) effected each of them differently. Additionally, the control variables in our statistical models, selected for their known potential effects on cognition (sex, age, BMI, psychiatric problems), only seldom reached statistical significant effects. This indicates that the smoking and SES effects were the predominant factors affecting performance. Therefore, our data set does not confirm the hypothesis that poor cool executive functions in low SES individuals (those whose parents had completed fewer years of education) leads to impaired control of emotions and impulses [71]. We also did not corroborate the idea that performance on tasks that tap hot and cool self-regulation abilities can be equated because they all involve conflict resolution [78]. Indeed, our finding support the ample evidence that self-regulation in the presence and absence of emotionally salient conflict differs. These abilities also vary in terms of developmental trajectories and brain activation, among other aspects, although they may interact in some scenarios (see [20]).

The measure selected to show the effects of SES on cognition was average parents' years schooling because this variable explained 21% of the variance in general knowledge (Information test; medium effect size). General knowledge is regarded as a proxy for prior cognitive stimulation that reflects many factors including quality of schooling (see [21,107,108]), and which explains a good part of children’s cognitive performance [138]. Fathers’ schooling had similar effects, but in developing nations children are often raised by their mothers alone. Hence, the average measure of parental schooling was regarded as the best variable to allow replication of our data in countries with wide socioeconomic variability. If we had used a composite score of all SES measures, interpretations of beta coefficients would have been difficult. The other SES measures also had lower, but similar effects, thus showing the multidimensional nature of the SES construct, which is influenced by several inter-correlated family-related and environmental experiences [72,73]. The exception was family income, which is a volatile measure of SES and represents current states [139] and not necessarily participants’ cognitive stimulation in their past, while they were growing up.

Lower average parental schooling, our SES indicator, was associated with poorer performance on all three separable cool executive domains proposed by Miyake et al. [28] (Shifting, Updating and Inhibition, all with small effect sizes) and Planning (medium effect size), reinforcing similar findings in studies with underprivileged children (see [71,76,74,77,140]). All these affected measures may be classified as cool skills since it is difficult to attribute socioemotional processing to the Stroop (Inhibition) and Zoo Map (Planning) tasks.

Although all our sample had graduated high school and was thus not composed of individuals in extremely socio-cultural vulnerable conditions, SES explained 22% of variance in Working Memory Capacity, mirroring findings in disadvantaged youngsters (see [76]). This cognitive measure is important for successfully dealing with interference (see [38,141,142]) and problem solving [19,38]. The absence of effects of mean parental schooling (SES) on hot skills, however, does not support claims that Working Memory Capacity impacts emotional regulation [19,38], or sporadic [79] and inconsistent [80,81,143] reports of a negative relationship between SES and hot self-regulation. General knowledge also mediated the effects of mean parental schooling (SES) on Working Memory Capacity, suggesting that low cognitive stimulation throughout one’s lifetime plays a part in impairing this domain. Contrariwise, effects of mean years of parents’ schooling on Planning were not mediated by Working Memory Capacity, general knowledge, nor a measure that represents the common factor of executive functioning (Inhibition). This suggests that higher-order planning difficulties in those whose parents had little chance to study (low SES) are not secondary to other executive problems, but rather show a specific SES-related effect. In contrast, parental schooling (SES) did not affect Access to Long-Term Memory (fluency), which may be more dependent on participants’ schooling (e.g. [144]) than parents’ education, nor Dual Tasking, which has not been specifically studied in relation to SES.

Our finding that being from a disadvantage family, as measured by parental years of schooling, was associated with impairment only on cool measures does not confirm the suggestion that impulsiveness in lower-SES individuals predisposes them to become smokers and/or diminishes their ability to quit [14,71], which could explain the higher prevalence of smokers among underprivileged people [2–4]. Difficulty in inhibiting automatic, non-affective responses found in low-SES individuals also fails to account for the higher prevalence of smokers in lower social strata. This is so because if this type of Inhibition and smoking were associated [14,71], we would have found a disinhibited profile in smokers irrespective of their parents’ education (SES), which was not the case here or in other studies that used similar measures [9,7]. This may be explained by the tendency in the literature to regard “Inhibition” as an assortment of hot skills, disregarding their possible fractionation. Indeed, the hot ability that is possibly associated with smoking may reflect another type of hot/impulsivity skill. Our data suggest that smoking is in fact related to aversion to waiting for incentives (Delay Discounting), as found here and in many other studies ([56,6,59,60,145,61] although, for the opposite result, see [8]), and greater self-assessed Impulsiveness, observed in the BIS-11 (total score [146], BIS-11 motor-impulsivity [6,8] and BIS-11 non-planning [6]). These hot effects were observed despite the absence of differences in mood (VAMS) between smokers and non-smokers. However, smokers showed higher ratings in the SRQ, which is expected as psychiatric symptoms have been associated with smoking initiation (see [147]) and have also been found to be a consequence of smoking [46].

Together with the absence of smoking-induced impairment in Inhibition, smoking did not impair Risk Taking (evaluated by the BART), thus corroborating most studies that have assessed this domain with the same [66,63,64], or similar tasks (Iowa Gambling Task [8,65]). Higher risk taking propensity in smokers has been found in a couple of studies, but other risk taking metrics were not altered in the same investigations (e.g. [7,62]), suggesting that this hot domain is not sensitive to smoking.

Therefore, it appears that the type of hot skill associated with smoking has to do with the difficulty of resisting immediate temptations [65]. This is consistent with: a) self-assessed impulsivity measured by BIS-11 (large effect size), as smokers may ignore possible negative consequences of smoking, which are abstract and distant, as opposed to the pleasure of smoking in the present moment; and b) preference for immediate reinforcements, assessed by Delay Discounting (medium effect size). Both of these types of hot skills contrast with changes in motivation in relation to cues with different affective values, which are assessed on immediate risk taking tests such as the BART, Iowa Gambling or equivalent laboratory measures; this difficulty also differs from having difficulty inhibiting automatic, non-affective responses (see [65]). The finding that smoking differently affects the four hot self-regulation domains studied here supports the idea that they are distinct constructs. In spite of our findings, there seems to be an association between long-term smoking and risk taking (e.g., [148]), possibly because all hot functions share some variance that may be captured in people who smoke more or have smoked for longer than our sample.

Overall, our findings concur with those of Conti et al.’s recent meta-analysis [149] in that hot cognition (what was termed “cognitive impulsivity”) is more strongly affected by chronic smoking than cool cognition. However, these authors also found effects favoring non-smokers in some cool domains obtained by pooling various types of tasks, which could have been due to the larger sample but also to confounding effects of age, education and SES, which their analysis was unable to control for.

What is still unclear is whether this worse hot self-regulation profile precedes or is a consequence of smoking [14]. Since none of the studied tobacco/nicotine exposure parameters mediated the effects of smoking on the affected hot domains (BIS-11 and Delay Discounting measures), we have confirmed previous claims that lifetime nicotine exposure (measured by dependence on nicotine and by cigarettes smoked) does not seem to be the cause of worse self-regulation, although these studies focused on cool domains [7,9,67] (but see [150] for an association of dependence on nicotine and speed, which was controlled in our cost measures). We extend these findings by showing that hot self-regulating impairment in young smokers is not mediated by cotinine concentrations and exhaled carbon monoxide, which were evidently higher in smokers [151–153] and correlated with smoking habits, as found by Fried et al. [5].

The absence of effects of these possible mediators on the effects of smoking on self-regulation might, at first glance, support the idea that cognitive problems do not increase with cigarette exposure, but may rather predate smoking, and/or cause smokers to have difficulty quitting once they begin smoking (see [9,14,67,69]). Some studies confirm this assumption by showing that self-reported trait impulsivity of a nonspecific type is associated with the onset of smoking [69]. Interestingly, these latter findings occurred regardless of educational level of participants and their parents, variables which are known to be related with SES, thus corroborating our absence of interaction between SES (as measured by parental schooling) and smoking on self-regulation.

Another hypothesis for the absence of association between measures of tobacco toxicity and self-regulation is that initial chronic exposure to nicotine is sufficient to cause neural alterations related to sensitivity to rewards [84], which may not necessarily increase thereafter even if people continue to smoke. In our case, it is possible that this threshold had already been surpassed among smokers and was sufficient to cause neuroadaptations that impaired hot self-regulation (see [84,154]) related to obtaining more immediate rewards (Delay Discounting) and difficulty in evaluating negative consequences of one’s actions (BIS-11).

Regarding cool self-regulation in smokers, it is difficult to compare our results to the literature. Prior studies either focussed on acute effects of nicotine, did not control for abstinence from nicotine, evaluated older participants in whom cumulative exposure to tobacco and/or age could influence results differently from our sample or, in particular, did not control for any measure of SES, which was found here to influence performance in most cool domains when assessed by parents’ education. As mentioned above, smokers tend to be from lower social strata so this could explain many of the reported smoking-induced effects on cool functions. It follows that conclusions should not be drawn about the extent to which cool performance is affected by chronic smoking itself without controlling for socioeconomic factors.

Together with unimpaired Stroop task performance found here and by other studies, which, as explained above, better reflects cool Inhibition, the absence of effects on Verbal Fluency corroborates findings reported by Wagner et al. [9]. Regarding Shifting, our finding confirms that chronic exposure to different nicotine doses does not affect this ability in mice [155] or in smokers with obsessive compulsive disorder in relation to non-smoking patients with the same disorder [156]. However, some studies found impaired Shifting in older, middle-aged smokers [157,158], while others did not [159]. Regarding Updating, we found no effects of smoking, thus adding more conflicting data to the literature. Ashor [160] and Flaudias et al. [159] reported impaired n-back performance in highly nicotine-dependent smokers, but did not state time since the last cigarette, so their data may have reflected effects of nicotine abstinence or its acute effects. Impairment on a verbal Updating task—but not on a visual one- was also found in another study [161], possibly due to the verbal/auditory problems associated with smoking (see [5]) and not Updating *per se*, which is supposed not to differ within sensory modalities (e.g. [162]). Contrary to our findings, smoking was associated with impaired Planning in the Tower of London and Tower of Hanoi tasks [7,67], but these tests should not be conceptualized as ‘planning’ tasks per se, despite this prevalent conception [28]. Smoking-induced impairment in Working Memory Capacity was not found here, but was shown to occur in the only two studies that used complex span tasks [7,161]. Again, however, these studies did not control for SES, which had a medium effect size here and may have accounted for these effects. Dual Tasking in smokers has not been previously investigated, so we have no data to confirm the absence of a smoking effect in this domain. Consequently, the evidence in the literature does not suffice to draw a firm conclusion as to the relations of chronic smoking with shifting, updating, planning and dual tasking, which should be further investigated. Differently, there seems to be a good indication that Inhibition as measured by the Stroop task and semantic verbal fluency are not impaired in chronic smokers.

Our findings were based on a small sample and are restricted to the effects of smoking on young people who had smoked at least 10 cigarettes per day for at least two years, were in optimum cognitive condition, had at least 11 years schooling, were free of most acute nicotine effects and were unaffected or only minimally affected by nicotine deprivation, and to females during the first few days of their menstrual cycle, since hormonal status alters self-regulation [90–92] and may influence the central effects of stimulants [94]. We can therefore not exclude the possibility that results would have differed had we tested more individuals, other populations, or used other tasks, although we did select measures from the literature that are representative of the self-regulation domains investigated. Exposure to tobacco did not mediate smoking-induced behavioural effects, but given the cross-sectional design of the present study, we are unable to say whether the relationship between smoking and higher self-reported impulsivity and greater preference for immediate reinforcement predate or follow chronic exposure to tobacco.

## Conclusions

We conclude that smokers who are underprivileged, as assessed by parental schooling, do not seem to be more susceptible to impaired self-regulation associated with smoking. This can be explained by the different profiles of smoking and SES effects: with the exception of Semantic Verbal Fluency and Dual Tasking, all cool self-regulation measures were sensitive to mean parental schooling (SES) alone, while hot skills were only influenced by smoking status. Specifically, smoking was related to difficulty in resisting immediate temptations and predicting future consequences of one’s actions, but not to general risky behaviour or impaired inhibition that did not involve socio-affective stimuli or responses. Targeting young smokers with intervention to quit or reduce smoking is essential to reduce morbidities and mortality [82], so strategies devised for this purpose should focus on improving these two hot domains, regardless of smokers’ SES. The fact that having parents who attended school for less years was negatively associated with cool skills, even if participants themselves had graduated high school, suggests that more access to schooling should counteract this impairment, but the effects may take at least a generation to show up.

## Supporting information

S1 Dataset (XLSX)
